# Parathyroid Hormone-Related Protein Promotes Rat Stem Leydig Cell Differentiation

**DOI:** 10.3389/fphys.2017.00911

**Published:** 2017-11-13

**Authors:** Tiantian Song, Yiyan Wang, Huitao Li, Lanlan Chen, Jianpeng Liu, Xianwu Chen, Xiaojun Li, Xiaoheng Li, Linxi Li, Qingquan Lian, Ren-Shan Ge

**Affiliations:** ^1^Department of Anesthesiology, Second Affiliated Hospital and Yuying Children's Hospital of Wenzhou Medical University, Wenzhou, China; ^2^Center of Scientific Research, Second Affiliated Hospital and Yuying Children's Hospital of Wenzhou Medical University, Wenzhou, China

**Keywords:** Leydig cell, stem Leydig cell, PTHrP, differentiation, testosterone

## Abstract

The regulatory factors for stem Leydig cell development are largely unknown. Herein, we reported that parathyroid hormone-related protein (PTHrP) may be a factor to regulate this process. The effects of PTHrP on rat stem Leydig cell proliferation and differentiation were investigated using a stem Leydig cell culture system and an ethane dimethane sulfonate (EDS)-treated *in vivo* Leydig cell regeneration model. PTHrP (1,000 pg/ml) significantly increased medium testosterone level and up-regulated STAR, CYP17A1, and 17β-HSD3 expressions. Co-treatment with PKA inhibitor H-89 or PKC inhibitor U73122 reversed PTHrP-mediated increase of testosterone production *in vitro*. Intratesticular injection of PTHrP (100 ng/testis) into the Leydig cell-depleted testis from post-EDS day 7 to 21 significantly increased serum testosterone level, up-regulated LHCGR, SCARB1, CYP11A1, 11β-HSD1, and CYP17A1 expressions. It also enlarged Leydig cell size without affecting PCNA-labeled Leydig cell number. This indicates that PTHrP promotes stem Leydig cell differentiation. PTHrP *in vivo* increased CREB and p-CREB levels, suggesting that PTHrP acts via a PKA-CREB signaling pathway. In conclusion, PTHrP stimulates stem Leydig cell differentiation without affecting its proliferation, showing its novel action and mechanism on rat stem Leydig cell development.

## Introduction

Leydig cells secrete testosterone, which is critical for maintaining the secondary sexual characteristics and promoting spermatogenesis in the adult male (Ye et al., 2017). The Leydig cell develops from the stem Leydig cell (Ye et al., 2017). Conceptually, pubertal Leydig cell development in rats is divided into four stages: stem (whole life-span), progenitor (postnatal day 14–21), immature (postnatal day 28–35), and adult Leydig cells (after postnatal day 56) (Ye et al., 2017). In the rat, a Leydig cell-depleting drug, ethane dimethane sulfonate (EDS), can specifically eliminate Leydig cells in the adult testis. Four days after treatment of EDS, all Leydig cells disappeared, and 21 days later progenitor Leydig cells emerged, indicating that a pool of stem Leydig cells can differentiate into the Leydig cell lineage for its regeneration (Teerds et al., 1988; Guo et al., 2013). The regenerated rat Leydig cells had the same identity as those found during puberty (Guo et al., 2013). Stem Leydig cells have the capability of self-renewing, homing to the niche, and committing into the Leydig cell lineage (Chen et al., 2017). Indeed, stem Leydig cells were purified from neonatal rat testis (Ge et al., 2006) or EDS-treated adult rat testis (Stanley et al., 2012) and they had the stem cell properties. When stem Leydig cells enter the Leydig cell lineage, they develop the steroidogenic machinery, including the signaling regulatory unit—luteinizing hormone (LH) receptor (LHCGR), the cholesterol transporting unit—high-density lipoprotein receptor (SCARB1), and steroidogenic acute regulatory protein (STAR), as well as the androgen biosynthesis unit—cytochrome P450 cholesterol side chain enzyme (CYP11A1), 3β-hydroxysteroid dehydrogenase 1 (3β-HSD1), cytochrome P450 17α-hydroxylase/20-lyase (CYP17A1), and 17β-hydroxysteroid dehydrogenase 3 (17β-HSD3). Interestingly, a glucocorticoid metabolizing enzyme, 11β-hydroxysteroid dehydrogenase 1 (11β-HSD1) begins expression in the advanced stage of Leydig cells (starting on postnatal day 28 or post-EDS day 28 in the rat) to protect Leydig cells from the detrimental effects of excess glucocorticoids (Phillips et al., 1989; Ge et al., 1997).

Although the identity of stem Leydig cells are reported in several species, including rats (Ge et al., 2006; Stanley et al., 2012; Li et al., 2016) and mice (Jiang et al., 2014), the regulatory factors are largely unknown. In a recent study, we setup a culture system of stem Leydig cells on the surface of seminiferous tubules to identify several growth factors that promote stem Leydig cell proliferation (platelet-derived growth factor AA and BB, Dessert Hedgehog, and fibroblast growth factor 2) and differentiation (platelet-derived growth factor AA, Dessert Hedgehog, and androgen; Li et al., 2016). In search of the regulatory factors, we checked the gene expression microarray data from mouse developing testis and ovary (GPL6246) and found that interstitial cells had much higher expression level of parathyroid hormone-related protein (PTHrP) receptor (*Pth1r*) than other testicular cells and that testicular cells including Sertoli and Leydig cells had PTHrP expressions although exact amount of PTHrP in the testis is still unclear (https://www.ncbi.nlm.nih.gov/geo/query/acc.cgi?acc=GPL6246). This suggests that PTH1R ligand PTHrP may be a critical factor to regulate Leydig cell development.

PTHrP is the endogenous ligand of PTH1R (Mannstadt et al., 1999). It is a secretory protein widely expressed in many tissues (Mannstadt et al., 1999). It contains certain homology with parathyroid hormone (PTH) (Okazaki et al., 2008). PTH1R is a member of the B subfamily of the G protein coupled receptor superfamily. PTHrP binds to PTH1R and activates two signal transduction pathways of intracellular adenylate cyclase-cyclic adenosine monophosphate-protein kinase A (PKA) pathway and phospholipase C (PLC)-cytoplasmic calcium-protein kinase C (PKC) pathway after binding to the receptor. PTHrP plays several roles in cell proliferation and differentiation. For example, PTHrP can promote the proliferation of osteoblast or immature osteoblast-like cell and promote the bone formation (Martin and Tremblay, 2005). However, the role of PTHrP in Leydig cell development is largely unknown. PTHrP is mainly expressed in Sertoli cells in rat fetal and neonatal testes, and it is primarily expressed in Leydig cells in adult testes (Campos et al., 1991). In the present study, we provided the direct evidence to support PTHrP-mediated stem Leydig cell differentiation both *in vitro* and *in vivo*.

## Materials and methods

### Chemicals and kits

PTHrP was purchased from PeproTech (Cat. no. 96-100-09-100; MW, 9.8 kDa; Rocky Hill, NJ). Immulite2000 Total Testosterone kit was purchased from Siemens (Germany). Culture medium (M199, DMEM/F12) and Click-iT EdU (EdU) imaging kit were purchased from Invitrogen (Carlsbad, CA). EDS was purchased from Pterosaur Biotech Co (Hangzhou, China).

### Animals and treatments

Adult (60-day-old) male Sprague Dawley rats were purchased from Shanghai Laboratory Animal Center (Shanghai, China). After 1-week adjustment, each rat was intraperitoneally injected EDS (75 mg/kg body weight/once), which was dissolved in a mixture of DMSO: H_2_O (1:3, v/v), to eliminate all Leydig cells from rat testis. For experiments *in vitro*, testes were collected 4 days after EDS injection (Kerr et al., 1985; Molenaar et al., 1985). After decapsulation of the testis, the seminiferous tubules were separated from the interstitium as previously described (Stanley et al., 2012). For experiments *in vivo*, after EDS treatment, rats were randomly divided into three groups (six rats per group), in which each rat daily received intratesticular injection (20 μl) of normal saline (control), 10 (~1 nM, assuming a testis volume of 1 ml), or 100 (~10 nM) ng/testis PTHrP for 14 days, starting on post-EDS day 7. Considering that there was increased number of macrophages during the first 2 days after EDS (Gaytan et al., 1995), we selected the post-EDS day 7 as the first injection day of PTHrP. Doses of PTHrP were selected based on the effective concentrations in a previous study, in which 0.1, 1, 10, and 100 nM PTH were used (Zhang et al., 2014).

On post-EDS day 21 when progenitor Leydig cells emerged in normal testis (Kerr et al., 1985; Molenaar et al., 1985), rats were euthanized and the blood samples were collected. Blood samples were centrifuged at 1,500 × g for 10 min to collect serum. The serum sample was stored at −20°C until testosterone and LH analysis. One testis per rat was collected and frozen in the liquid nitrogen for mRNA and protein expression study. The contralateral testis was punched three holes using a G27 syringe needle and fixed in Bouin's solution. All animal procedures were performed in accordance with the protocols approved by Animal Care and Use Committee of Wenzhou Medical University.

### Isolation and culture of seminiferous tubules

Seminiferous tubules were isolated as previously described (Li et al., 2016). They were cultured in a basal medium (BM), which had M199/F12 (1:1, v/v) medium supplemented with 0.1% BSA, 15 mM HEPES, 2.2 mg/ml sodium bicarbonate, and penicillin/streptomycin (100 U/ml and 100 μg/ml), in a humidified atmosphere of 5% CO_2_ at 37°C for a week. They were cultured in BM with addition of 5 ng/ml LH (LH), 5 mM lithium chloride (LI), insulin/transferrin/selenium (ITS), or LH, LI, and ITS together (LH+LI+ITS) for additional 2 weeks. After that, the ability of Leydig cells on the surface of the seminiferous tubules to produce testosterone was determined as previously described (Li et al., 2016).

### EdU incorporation into stem Leydig cells for proliferation assay

Stem Leydig cell proliferation was measured by the EdU Alaxa Fluor Kit (Life Technologies, USA) according to the manufacturer's instructions. In brief, the seminiferous tubules were cultured in BM as above and treated with 0–1,000 pg/ml PTHrP for 5 days. Then, an aliquot (2 μl) of 1:1,000 diluted EdU was added to the well and incubated for 24 h. Seminiferous tubules were washed twice with 500 μl PBS buffer containing 3% bovine serum albumin. The tubules were then fixed in 500 μl 4% paraformaldehyde at room temperature for 30 min. Tubules were washed and incubated with the reaction solution in the dark for 45 min. The tubules were washed again and mounted on a slide for visualization under a fluorescence microscope (Olympus, Japan) and images were captured. EdU-positive cells on the surface of the tubules were counted using the ImageProPlus 7.0 software (Media Cybernetics, Rockville, MD, USA).

### Stem Leydig cell proliferation and differentiation assay

To test the effects of PTHrP on the proliferation, different concentrations (10, 100, and 1,000 pg/ml) PTHrP were added to BM and cultured for 7 days and then the tubules were switched to the BM containing LH+LI+ITS for additional 2 weeks to induce the formation of Leydig cells to produce testosterone and then the medium testosterone levels were measured as described (Li et al., 2016).

For differentiation assay, the seminiferous tubules were cultured in BM for 7 days and then the tubules were switched to LH+LI+ITS medium containing different concentrations (10, 100, and 1,000 pg/ml) of PTHrP for additional 2 weeks to induce stem Leydig cell differentiation and then the medium testosterone levels were measured as described (Li et al., 2016). The PTHrP concentrations were selected based on the minimal effective concentration *in vivo*.

### Quantitative real-time PCR (qPCR)

Total RNAs were isolated from the seminiferous tubules and testes using a Trizol kit according to the manufacturer's instructions (Invitrogen, Carlsbad, CA, USA). The concentrations of RNAs were measured by reading OD value at 260 nm by NanoDrop 2000 (Thermo Scientific, Shanghai, China). The first strand of cDNA was synthesized and used as the template for qPCR as previously described (Zhang et al., 2013). SYBR Green qPCR Kit (Takara, Otsu, Japan) was used to analyze the mRNA levels (*Lhcgr, Scarb1, Star, Cyp11a1, Hsd3b1, Cyp17a1, Hsd17b3, Srd5a1*, and *Hsd11b1*). The PCR reaction mixture contained 7.5 μl SYBR Green mix, 1.5 μl forward and reverse primer mix, 0.02 μg diluted cDNA, and 4 μl RNase-free H_2_O. The procedure of qPCR was set as the following: 95°C for 5 min, followed by 40 cycles of 95°C for 10 s, and 60°C for 30 s. The house-keeping gene, ribosomal protein S16 (*Rps16*), was used as the internal control. The mRNA level of each gene was read as the Ct value and calculated using a standard curve method and was normalized to *Rps16* as previously described (Lin et al., 2008). The primers were listed in Supplementary Table 1.

### Serum and medium testosterone assay

Serum and medium concentrations of testosterone were measured by a solid-phase competitive chemiluminescent enzyme immunoassay using Immulite2000 Total Testosterone kit (Siemens, Germany) according to the procedure described by the manufacturer. Blank medium was measured as the background. The lower detection limit was 0.2 ng/ml. The intra-assay and inter-assay CVs were 5.75 and 7.53%, respectively.

### ELISA for serum LH levels

Serum LH levels were detected with an ELISA kit according to the manufacturer's instructions (Chemicon CA, USA). Briefly, 200 μl serum or medium samples and 50 μl assay solution were added to pre-coated 96-well plates. The plates were incubated for 2 h at room temperature, and washed five times with washing buffer. A peroxidase-conjugated IgG anti-LH solution (100 μl) was added into each well and the mixture was incubated for 2 h at room temperature. After plates were washed five times, 100 μl substrate buffers were added into each well, and the reaction mixture was incubated in the dark place for 30 min at room temperature. The enzyme reaction was stopped by 50 μl stop solution. The quantification of LH levels was obtained by a microplate reader at 550 nm with correction wavelength at 450 nm. Data was analyzed by GraphPad Prism software (Version 6, GraphPad Software Inc., San Diego, CA).

### Western blot analysis

Western blotting was performed as previously described (Wu et al., 2017). Tissue proteins were prepared from *in vitro* 2-week PTHrP-treated rat seminiferous tubules or *in vivo* 3-week PTHrP-injected testes. Tissues were homogenized and lysed with RIPA buffer (Bocai Biotechnology, China) to obtain protein samples. BCATM Protein Assay Kit (Takara, Japan) was used to measure the total protein concentrations of samples. A total protein (30 μg) each sample was added to the well of PAGE gel (10% w/v acrylamide) and electrophoresed and then the separated proteins were blotted onto the nitrocellulose membranes. The membranes were blocked with 5% non-fat milk in TBST buffer for 2 h and incubated with primary antibodies against LHCGR, CYP11A1, CYP17A1, 11β-HSD1, 3β-HSD1, or β-actin (ACTB) at 4°C overnight. After that, the membranes were washed and incubated with HRP-conjugated anti-rabbit or anti-goat IgG secondary antibodies (1:2,000, Bioword, USA) for 2 h at room temperature. The band was visualized by chemiluminescence using an ECL kit (Amersham, Arlington Heights, IL). The density of target protein was normalized to ACTB, the house-keeping protein, and the density was calculated using J-Software. All the antibodies used were listed in Supplementary Table 2.

### Immunohistochemistry and immunofluorescence

Testes and cultured seminiferous tubules were fixed with Bouin's solution (Sigma-Aldrich, St. Louis, MO), embedded in paraffin, and sectioned for morphological analysis. For immunohistochemistry, sections were stained with CYP11A1 and 11β-HSD1 according to standard protocols. Diaminobenzidine was used to visualize the antibody-antigen complexes with brown cytoplasmic staining of Leydig cells. For immunofluorescence, Ca^2+^ and Mg^2+^ free HBSS (0.5% BSA) were used to wash the cultured seminiferous tubules or sections. The primary antibodies of 3β-HSD1, α-SMA (SMA), CYP11A1, or PCNA were added and incubated for 60 min. Then, the fluorescent secondary antibody (Alexa-conjugated anti-rabbit or anti-mouse IgG, 1:500) was used after the primary antibody. Click-It EdU imaging kit was used to monitor cell divisions followed by the manufacturer's instructions. Using fluorescent microscopy to visualize the sections after counterstained with mounting medium containing DAPI.

### Counting Leydig cell number

To count CYP11A1-positive Leydig cell numbers or 11β-HSD1-positive immature Leydig cell numbers, sampling of the testis was performed according to a fractionator technique as previously described (Mendis-Handagama et al., 1989). Briefly, six testes per group per time point were used. Each testis was cut in eight parts and two parts were randomly selected. Then, parts were cut into four pieces and one piece was randomly selected from total eight pieces. These pieces of testis were embedded in paraffin in a tissue array. Paraffin blocks were sectioned in 6-μm-thick sections. Ten sections were randomly sampled from each testis per rat. Sections were used for immunohistochemical staining as above. The identification of all cells in the Leydig cell lineage was performed by staining with a polyclonal antibody specific for CYP11A1, and the identification of immature Leydig cells was performed by staining with a polyclonal antibody specific for 11β-HSD1 as above because this biomarker is present in Leydig cells at the advanced stages. Images were taken and total microscopic fields per section were counted. The total number of Leydig cells was calculated by multiplying the number of Leydig cells counted in a known fraction of the testis by the inverse of the sampling probability.

### Statistical analyses

Data was showed as the mean ± SEM. *P* < 0.05 was considered to be statistically significant. The difference between groups was evaluated by one-way ANOVA followed by *ad-hoc* Dunnett's multiple comparison to compare the difference between two groups. For Western blot analysis, we performed one-side paired student *t*-test followed by Sidak adjustment to identify the significance between treated groups and the control. The significant difference was set as ^*^P < 0.05, ^**^P < 0.01, or ^***^P < 0.001.

## Results

### PTHrP stimulates stem Leydig cell differentiation *in vitro*

**Figure 1:**
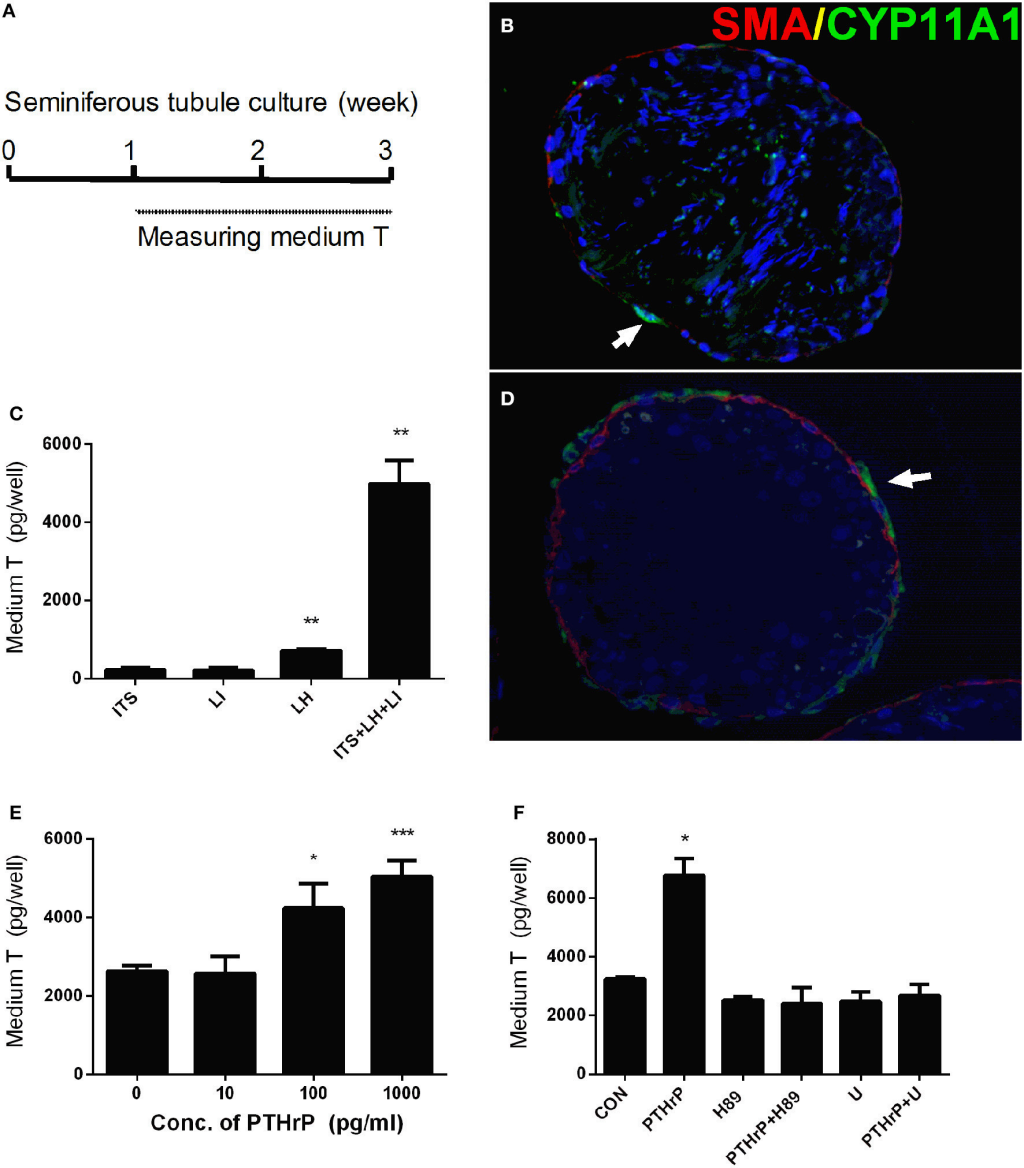
PTHrP induces stem Leydig cell differentiation *in vitro*. Scheme of stem Leydig cell culture for 21 days **(A)**. Staining of Leydig cells using CYP11A1 in the cross sections of the seminiferous tubules after 14 days in the ITS medium **(B)** and LH+ITS+LI medium **(D)**. α-SMA (SMA, red color) was used to label peritubular myoid cells and CYP11A1 (green color, white arrow) was used to label Leydig cells. Medium testosterone levels after 14 days in ITS, LH, LI, and ITS+LH+LI **(C)**, ITS+LH+LI together with various concentrations of PTHrP **(E)** as well as 1,000 pg/ml PTHrP with or without H-89 (1 μM) or U73122 (U, 1 μM) **(F)**. Mean ± SEM, *n* = 6, ^*^P < 0.05, ^**^P < 0.01, ^***^P < 0.001 when compared to the control.

We setup a Leydig cell-depleted seminiferous tubule culture system. Stem Leydig cells reside on the surface of the seminiferous tubules (Li et al., 2016). We treated the tubules with ITS, LH (5 ng/ml), LI (5 mM), or ITS+LH+LI for 2 weeks (Figure 1A). We labeled peritubular myoid cells with α-SMA (SMA, red color) and Leydig cells with CYP11A1 (green color). As shown in Figure 1B (ITS), only few Leydig cells were formed. However, ITS+LH+LI treatment induced many CYP11A1-positive cells on the surface of the tubules (Figure 1D). We measured the medium testosterone levels, and we found that ITS+LH+LI significantly increased medium testosterone levels (Figure 1C). This data suggests that ITS+LH+LI is the Leydig cell-differentiation inducer. We added different concentrations of PTHrP into ITS+LH+LI-containing medium and cultured stem Leydig cells for 2 weeks. PTHrP concentration-dependently increased medium testosterone levels (Figure 1E), suggesting that PTHrP promotes stem Leydig cell differentiation. To investigate the PTHrP-mediated signaling pathway, we used H-89 (a PKA blocker) and U73122 (a PKC blocker). Neither H-89 nor U73122 alone affected the medium testosterone levels (Figure 1F). However, H-89 and U73122 significantly reversed the PTHrP-induced increase of testosterone levels (Figure 1F). These data indicate that PTHrP promotes stem Leydig cell differentiation via both PKA and PKC pathways.

### PTHrP up-regulates Leydig cell specific gene and protein expressions *in vitro*

**Figure 2:**
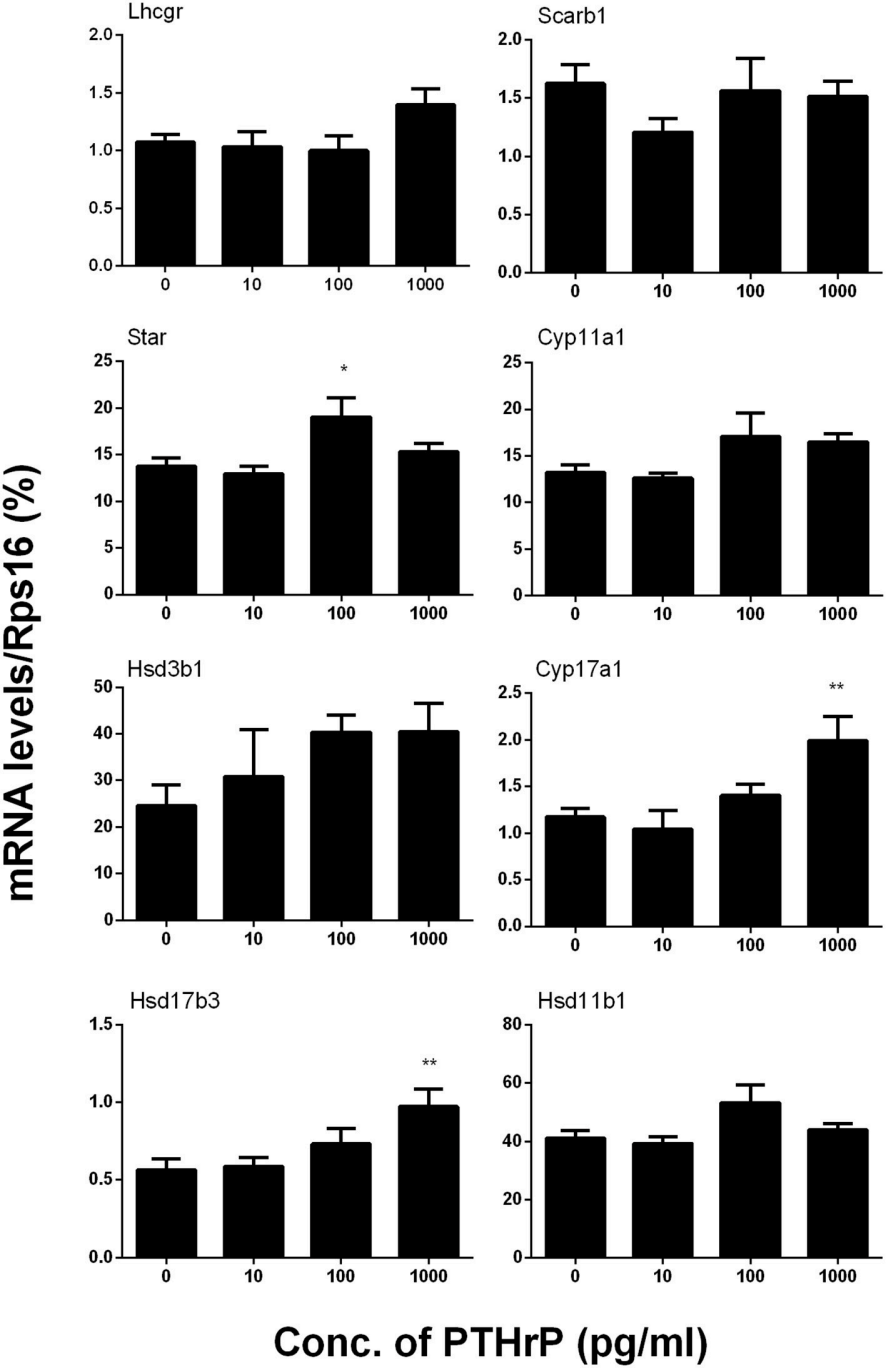
PTHrP up-regulates the expression levels of some Leydig cell-specific genes *in vitro*. The mRNA levels of *Lhcgr, Scarb1, Star, Cyp11a1, Hsd3b1, Cyp17a1, Hsd17b3*, and *Hsd11b1* were analyzed by qPCR in the seminiferous tubules treated with 0, 10, 100, and 1,000 pg/ml PTHrP for 14 days. Mean ± SEM, *n* = 6, ^*^P < 0.05, ^**^P < 0.01 when compared to the control.

**Figure 3:**
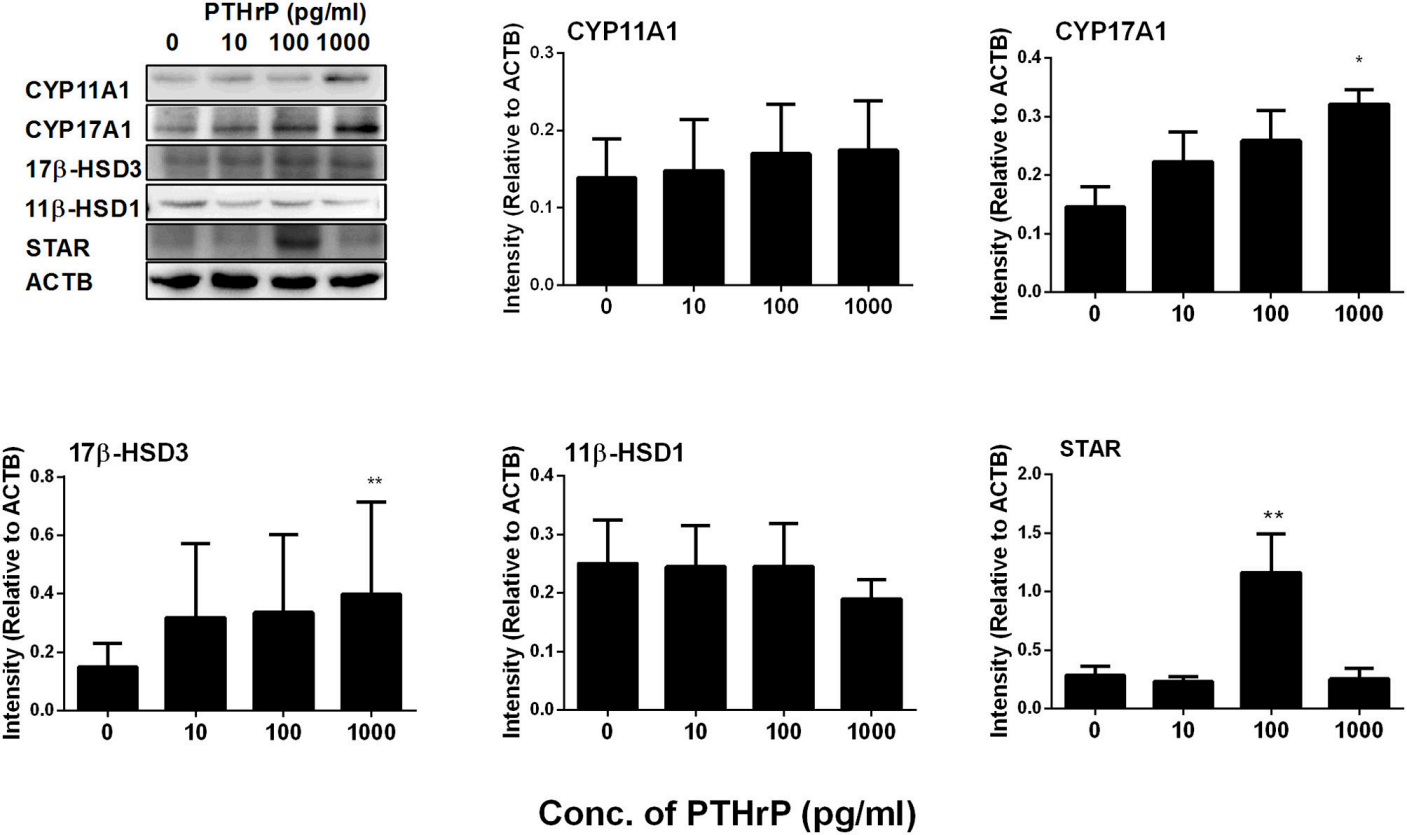
PTHrP up-regulates the expression levels of some Leydig cell-specific proteins *in vitro*. The protein levels of CYP11A1, CYP17A1, 17β-HSD3, 11β-HSD1, and STAR were analyzed by Western blot in the seminiferous tubules treated with 0, 10, 100, and 1,000 pg/ml PTHrP for 14 days. Mean ± SEM, *n* = 3, ^*^P < 0.05, ^**^P < 0.01 when compared to the control.

We further measured Leydig cell specific gene and protein expression levels during the differentiation. QPCR data showed that PTHrP up-regulated *Star* expression level at 100 pg/ml and increased *Cyp17a1* and *Hsd17b3* expression levels at 1,000 pg/ml (Figure 2), while it did not affect *Lhcgr, Scarb1, Cyp11a1, Hsd3b1*, and *Hsd11b1* mRNA levels (Figure 2). Indeed, Western blot confirmed that PTHrP significantly increased STAR level at 100 pg/ml and CYP17A1 level at 1,000 pg/ml (Figure 3). These results suggest that PTHrP promotes stem Leydig cell differentiation by up-regulating STAR and CYP17A1 expression.

### PTHrP does not influence stem Leydig cell proliferation *in vitro*

In order to test whether PTHrP regulated stem Leydig cell proliferation *in vitro*, the Leydig-cell-depleted seminiferous tubule culture was setup and EdU incorporation into stem Leydig cells was performed. Our previous study demonstrated that EdU incorporated the nuclei of peritubular cells (possible stem Leydig cells) outside myoid cells (Li et al., 2016). EdU was incorporated into some stem Leydig cell nuclei (Supplementary Figure 1A) on the surface of the seminiferous tubules. PTHrP treatment for 5 days did not increase the EdU incorporative rate, indicating that PTHrP does not influence stem Leydig cell proliferation *in vitro*. To further confirm this, we used an indirect approach, in which stem Leydig cells were treated with PTHrP for a week and then these stem Leydig cells were induced into the Leydig cell lineage by 2-week treatment of ITS+LH+LI. We assumed if PTHrP increased the numbers of stem Leydig cells, which were all induced into Leydig cells, thus increasing the medium testosterone levels. As shown in the Supplementary Figure 1, the medium testosterone levels did not change after PTHrP pretreatment, confirming that PTHrP does not promote stem Leydig cell proliferation.

### PTHrP promotes Leydig cell regeneration *in vivo*

**Figure 4:**
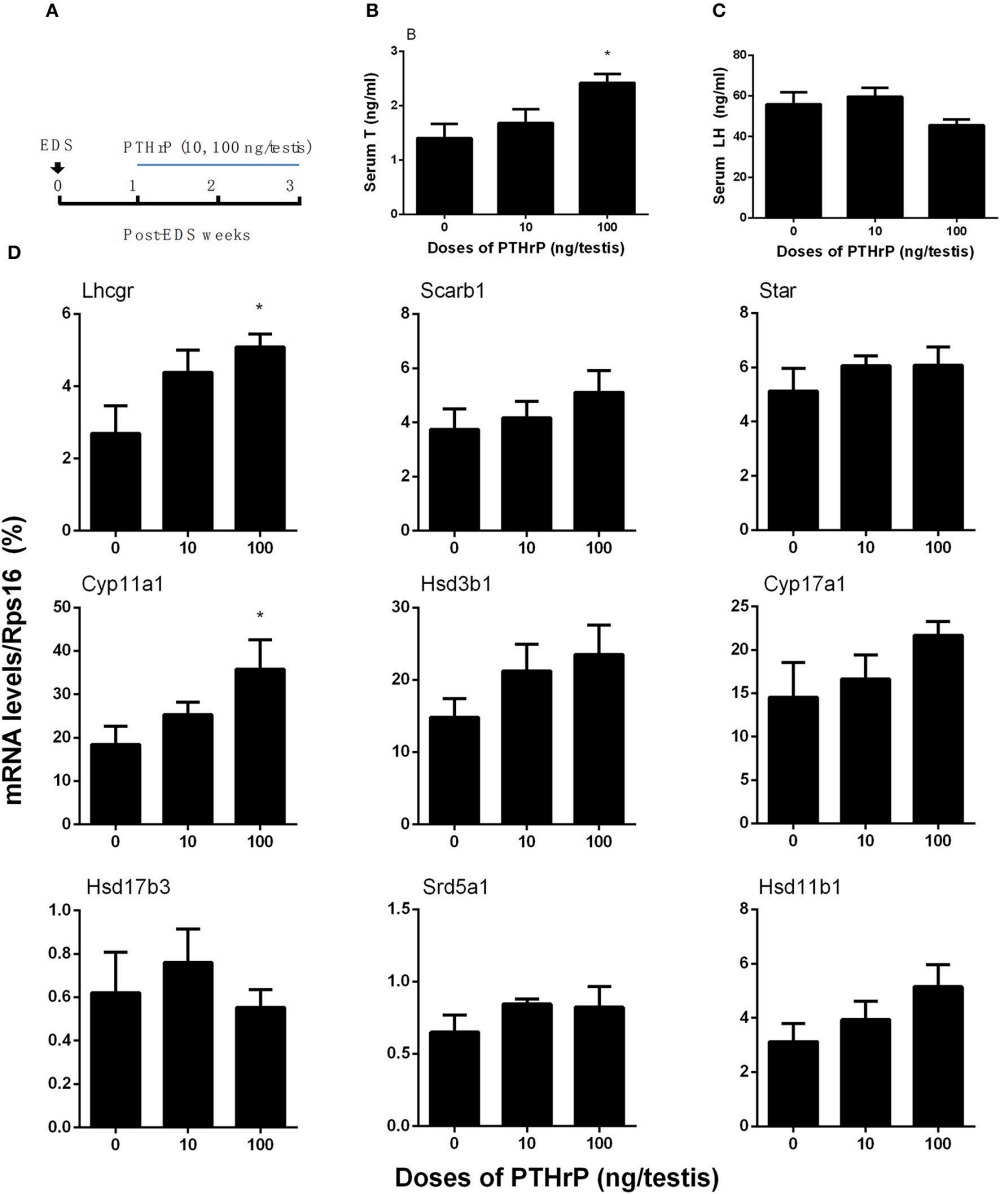
PTHrP induces stem Leydig cell differentiation *in vivo*. Scheme of PTHrP-treated Leydig cell regeneration for 14 days **(A)**. Serum testosterone levels **(B)** and LH levels **(C)**. The mRNA levels of *Lhcgr, Scarb1, Star, Cyp11a1, Hsd3b1, Cyp17a1, Hsd17b3, Srd5a1*, and *Hsd11b1* were analyzed by qPCR in the testes treated with 0, 10, and 100 ng/testis PTHrP for 14 days **(D)**. Mean ± SEM, *n* = 6, ^*^P < 0.05 when compared to the control.

**Figure 5:**
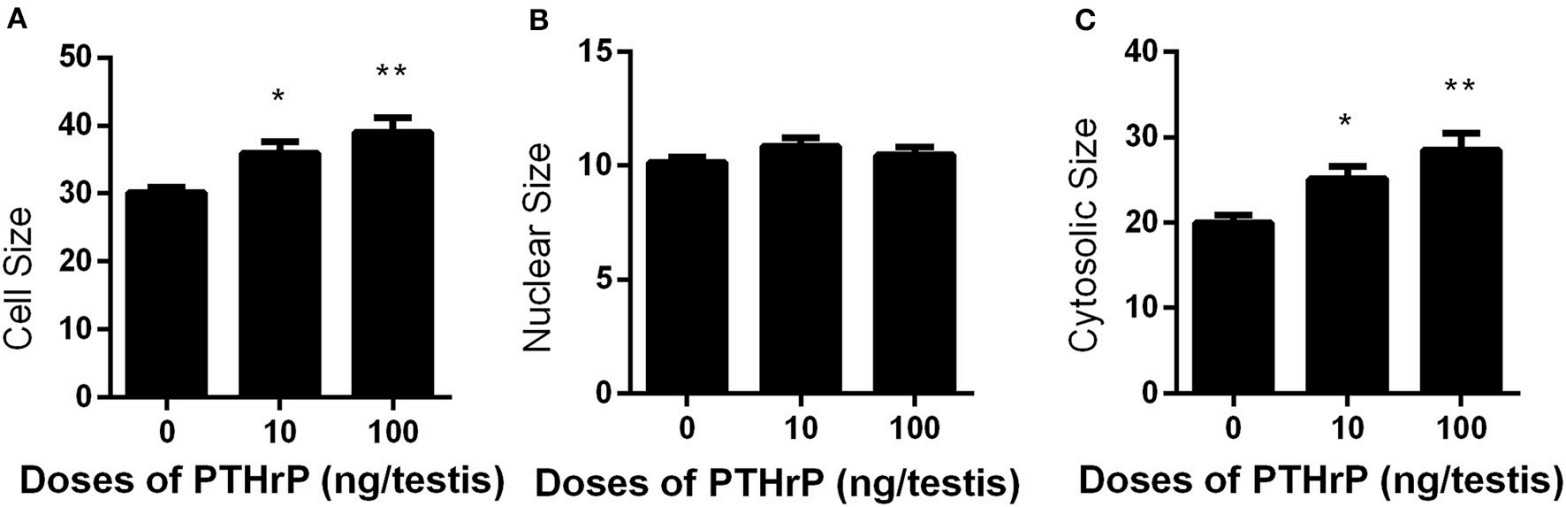
Effects of PTHrP on cell, nuclear, and cytoplasmic sizes of Leydig cells in the testes after *in vivo* treatment. Immunohistochemical staining of CYP11A1 of the testes from the rats treated with 0, 10, and 100 ng/testis PTHrP on post-EDS day 7 for 21 days. **(A–C):** cell, nuclear, cytoplasmic size, respectively. Mean ± SEM, *n* = 6. ^*^P < 0.05, ^**^P < 0.01 when compared to the control.

Seven days after EDS, all Leydig cells in the testis were eliminated, while stem Leydig cells were still present (Hu et al., 2010). After EDS treatment, rats were randomly divided into three groups (six rats per group). We daily injected PTHrP (0, 10, or 100 ng/testis) from post-EDS day to day 28, at which immature Leydig cells were emerged (Guo et al., 2013). The dose was selected based our *in vitro* PTHrP concentration (Figure 4A). PTHrP treatment did not alter rat body weight and testis weight (Supplementary Table 3). When compared to the control, PTHrP significantly increased serum testosterone level at 100 ng/testis (Figure 4B), while it did not affect LH levels (Figure 4C), indicating that the action of PTHrP was within the testis. We measured some Leydig cell specific gene expression levels, and we found that PTHrP up-regulated *Lhcgr* and *Cyp11a1* levels (Figure 4D). We stained Leydig cells using CYP11A1 to measure the Leydig cell size, cytoplasmic size, and nuclear size. As shown in Figures 5A–C, PTHrP dose-dependently increased Leydig cell and cytoplasmic size without affecting the nuclear size, indicating that these Leydig cells morphologically are more mature than the Leydig cells in the control.

**Figure 6:**
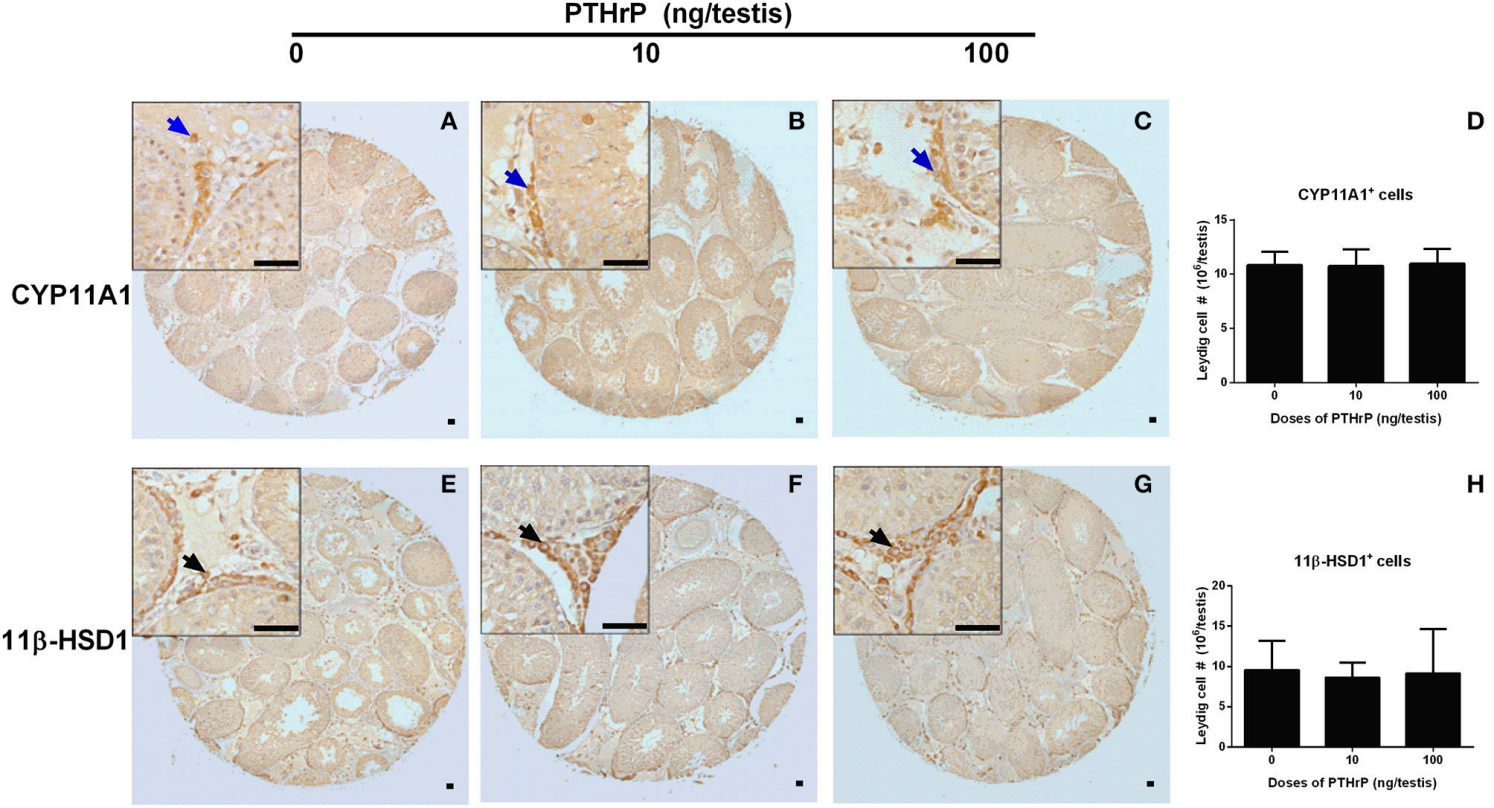
Morphology of Leydig cells in the testes after *in vivo* PTHrP treatment. Immunohistochemical staining of CYP11A1 **(A–D)** and 11β-HSD1 **(E–H)** of the testes from the rats treated with 0, 10, and 100 ng/testis PTHrP on post-EDS day 7 for 21 days. **(A,E)**: the control (0 pmol/testis PTHrP); **(B,F)**: 10 ng/testis PTHrP; **(C,G)**: 100 ng/testis PTHrP; **(D,H)**: quantitative data. Blue arrow indicates CYP11A1 positive Leydig cells. Black arrow indicates 11β-HSD1 positive Leydig cells. Bar = 50 μm. Mean ± SEM, *n* = 6. No significant difference between PTHrP group and the control was observed.

**Figure 7:**
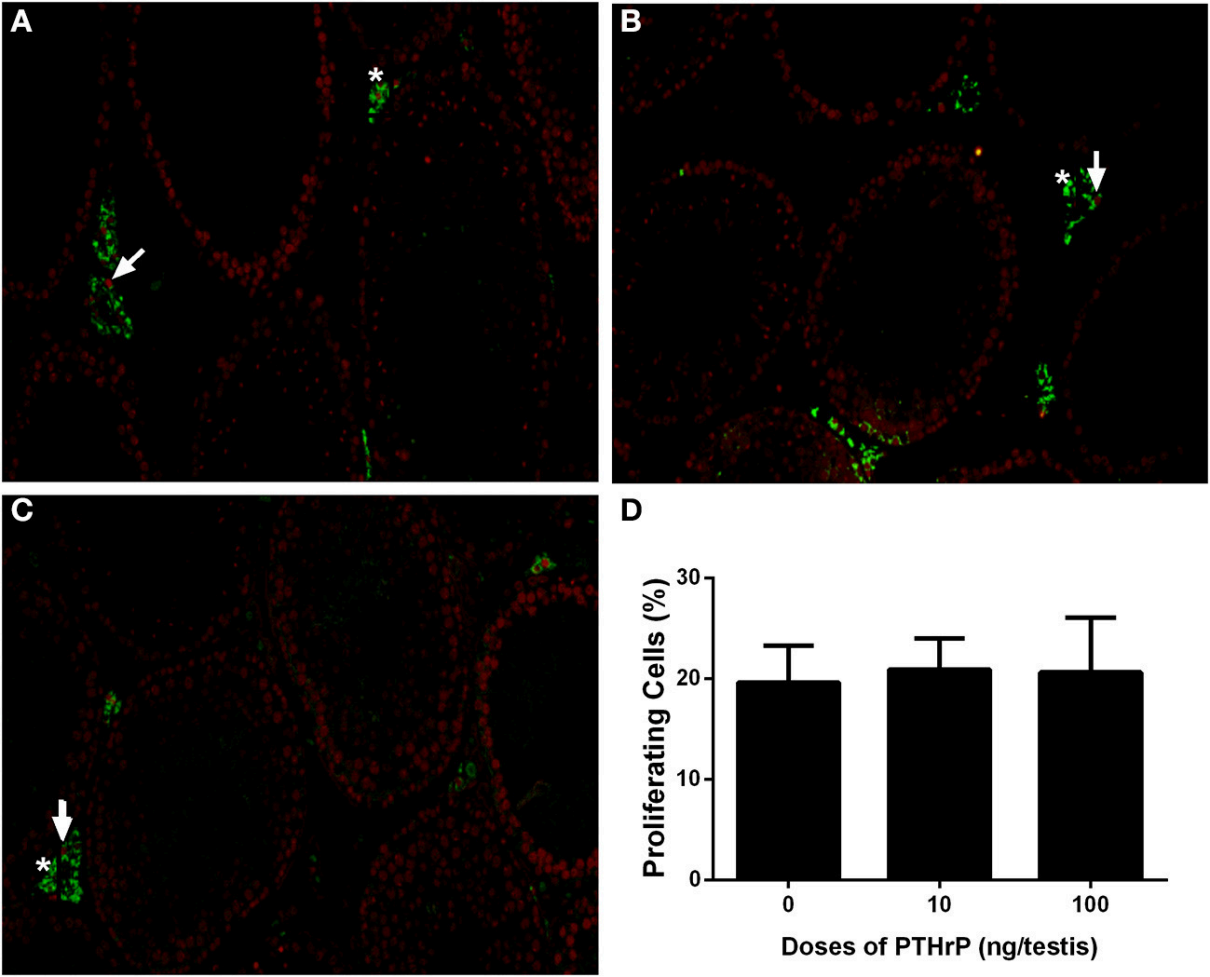
PTHrP does not affect proliferation of Leydig cells *in vivo*. Immunofluorescent staining of PCNA (red color in the nucleus) and 11β-HSD1 (green color in the cytosol) of the testes from the rats treated with 0 **(A)**, 10 **(B)**, and 100 **(C)** ng/testis PTHrP on post-EDS day 7 for 21 days. **(D)**: Quantitative data. White arrow indicates PCNA-positive Leydig cells. White “^*^” indicates PCNA-negative Leydig cells. Mean ± SEM, *n* = 6. No significant difference between PTHrP group and the control was observed.

We stained Leydig cells with CYP11A1 (all Leydig cells) and 11β-HSD1 (Leydig cells at the advanced stage) and counted whether PTHrP increased Leydig cell number. As shown in Figure 6, PTHrP did not increase the number of CYP11A1-positive or 11β-HSD1-positive Leydig cells. We further used PCNA to identify the proliferating cells and CYP11A1 to stain Leydig cells. As shown in Figure 7, double staining of PCNA and CYP11A1 showed that PTHrP did not increase the Leydig cell proliferative rate. These results indicate that PTHrP did not influence Leydig cell proliferation but stimulated differentiation.

**Figure 8:**
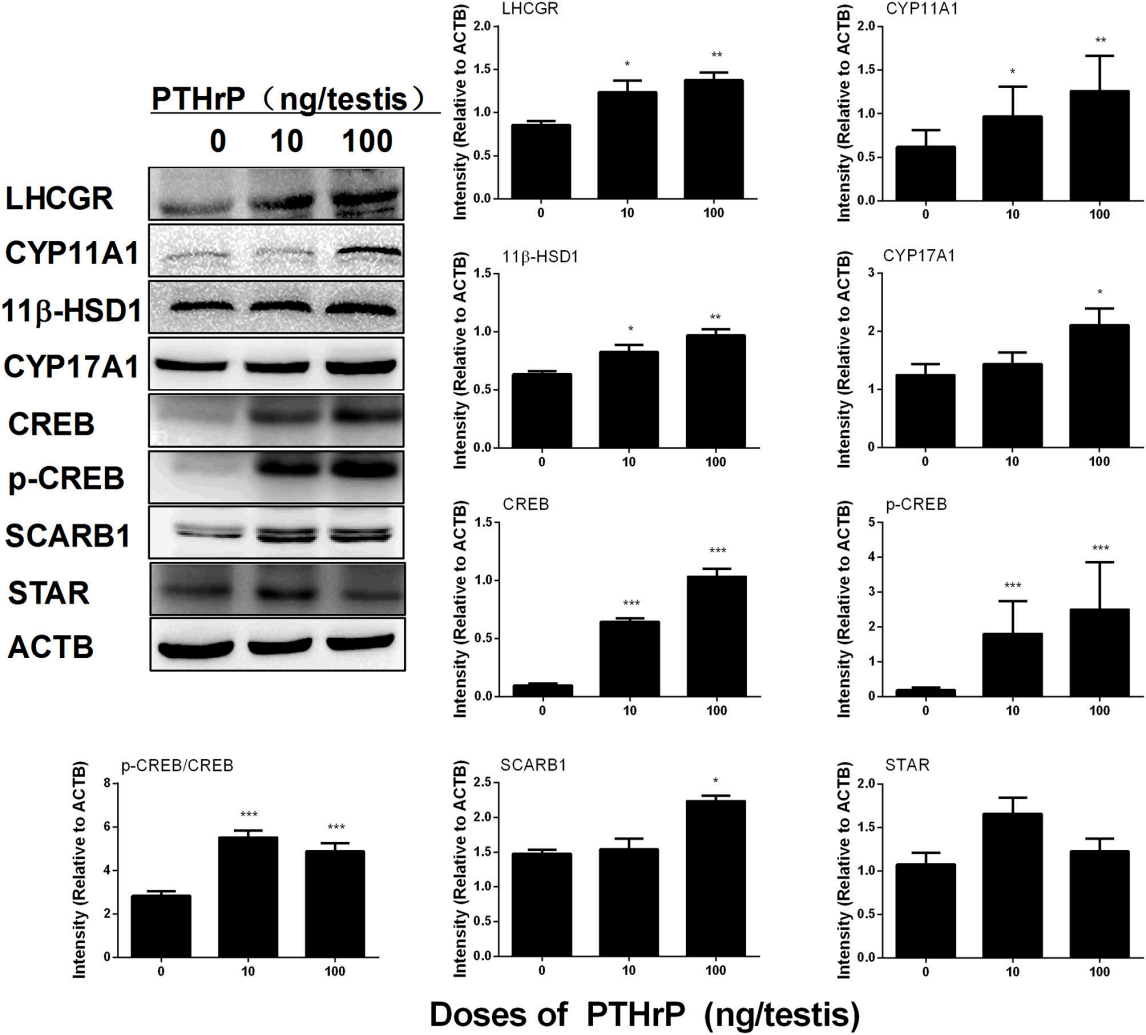
PTHrP affects Leydig cell-specific protein levels *in vivo*. Left panel: gel; Right panel: quantitative data. The protein levels of LHCGR, CYP11A1, CYP17A1, 11β-HSD1, CREB, p-CREB, CREB/p-CREB, SCARB1, STAR, and ACTB (internal control) were analyzed by Western blot in testes from the rats treated with 0, 10, and 100 ng/testis PTHrP on post-EDS day 7 for 21 days. Mean ± SEM, *n* = 4–5, ^*^P < 0.05, ^**^P < 0.01, and ^***^P < 0.001 when compared to the control.

**Figure 9:**
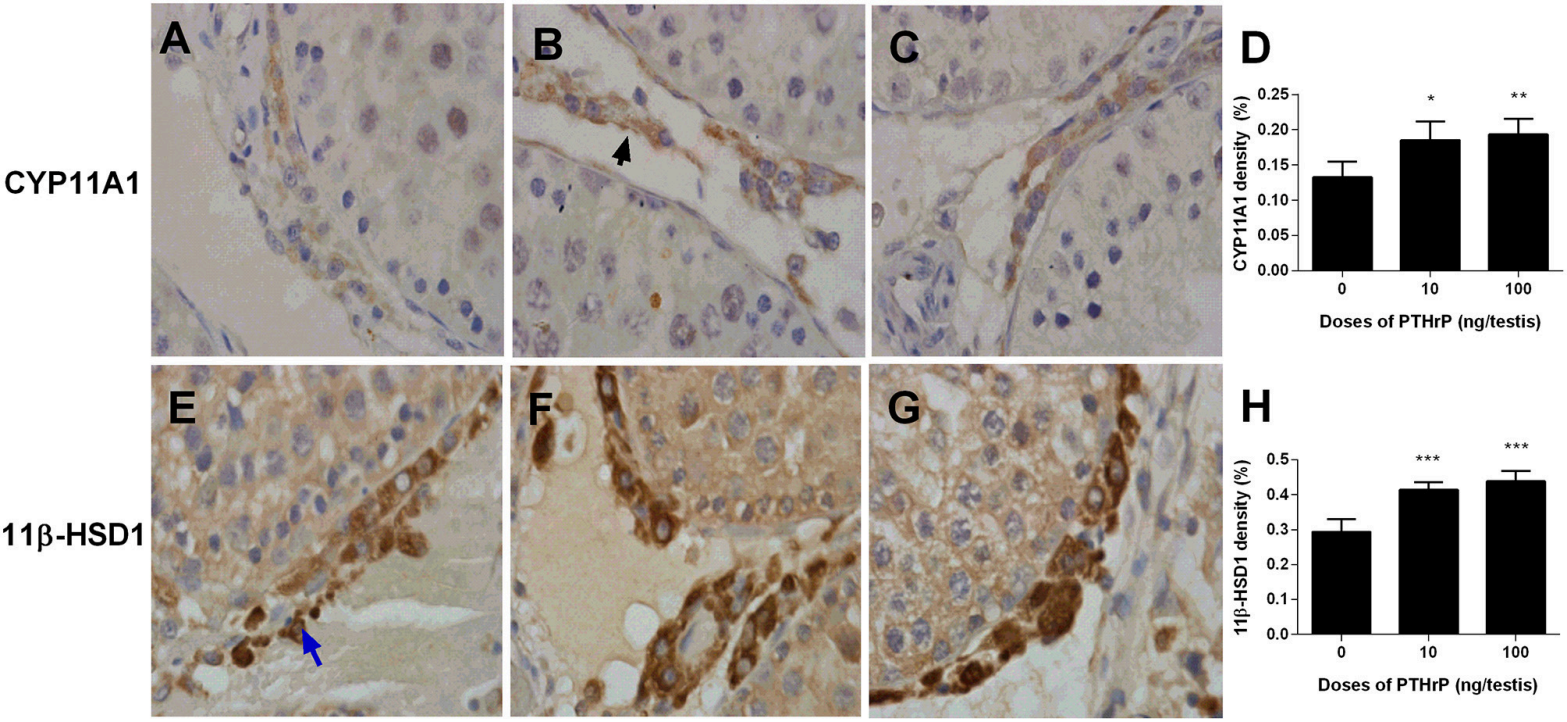
Semi-quantitative measurement of CYP11A1 and 11β-HSD1 levels of Leydig cells in the testes after *in vivo* PTHrP treatment. Immunohistochemical staining of CYP11A1 **(A–D)** and 11β-HSD1 **(E–H)** of the testes from the rats treated with 0, 10, and 100 ng/testis PTHrP on post-EDS day 7 for 21 days. **(A,E)** The control (0 PTHrP); **(B,F)** 10 ng/testis PTHrP; **(C,G)** 100 ng/testis PTHrP; **(D,H)** quantitative data. Black arrow indicates CYP11A1 positive Leydig cells. Blue arrow indicates 11β-HSD1 positive Leydig cells. Mean ± SEM, *n* = 6. ^*^P < 0.05; ^**^P < 0.01; ^***^P < 0.001 when compared to the control.

Western blot data showed that PTHrP increased LHCGR, CYP17A1, 11β-HSD1, and SCARB1 levels (Figure 8). We also used the semi-quantitative analysis of CYP11A1 and 11β-HSD1 densities in the testis tissue array to investigate the CYP11A1 and 11β-HSD1 protein levels in the individual Leydig cell (Figure 9). We found that PTHrP dose-dependently increased CYP11A1 and 11β-HSD1 protein levels in the individual Leydig cell. Since PTHrP may act via PKA-CREB signaling (Ko et al., 2001), we measured CREB and p-CREB levels using semi-quantitative Western blotting (Figure 8). Indeed, PTHrP significantly increased the levels of these two proteins. These data indicate that PTHrP increases stem Leydig cell differentiation via PKA-CREB signaling.

## Discussion

PTHrP is a heterologous polypeptide, which has definite homologous sequence with PTH at the end of the 13th residue (Harrington et al., 2007). PTHrP can be expressed in the skin, bone marrow, brain, cardiovascular, thyroid, parathyroid, bone, and testis, playing physiological roles in the local tissues by the way of paracrine or autocrine (Nikitovic et al., 2016). In rat testis, PTHrP is mainly secreted by Sertoli cells during fetal and neonatal periods, and it is mainly secreted by adult Leydig cells at the adulthood (Campos et al., 1991), suggesting that PTHrP can act by paracrine (Sertoli cells) and autocrine (Leydig cells) to regulate stem Leydig cell development. Herein, we identified a critical role for PTHrP, which promoted stem Leydig cell differentiation.

It was confirmed that PTHrP had three receptors, PTH1R, PTH2R, and PTH3R. PTH1R is the most common receptor as a protein-coupled receptors, which has the same affinity with PTH or PTHrP (Martin, 2005). PTHrP receptors are expressed in osteoblasts for bone formation during the embryonic development. Becher et al. (2010) demonstrated that the expression of PTH1R gradually declined with the progress of osteoarthritis. PTHrP plays an important role in the regulation of cartilage osteogenesis, as shown by the fact that it stimulated the proliferation of chondrocytes through PTH1R and inhibited chondrocytes differentiating into hypertrophic chondrocytes, and regulated cartilage osteogenesis (Miao et al., 2004).

The role of PTHrP in Leydig cell development is not clear until the present study, in which we demonstrated that PTHrP promoted stem Leydig cell differentiation without affecting its proliferation. Apparently, PTHrP *in vivo* increased the protein expression levels of LHCGR, SCARB1, CYP11A1, CYP17A1, and 11β-HSD1 in the testis. Although three protein (SCARB1, CYP17A1, and 11β-HSD1) levels were not correlated with their qPCR data, there was a clear increased trend for these genes. Since the CYP11A1-positive or 11β-HSD1 positive Leydig cell numbers were not changed, the up-regulated expressions of these proteins indicates that their expression levels per Leydig cells were increased. Indeed, our previous study demonstrated that the gene expression levels for these proteins were significantly increased during the course of Leydig cell regeneration (Zhang et al., 2015). Therefore, *in vivo* treatment of PTHrP could stimulate Leydig cell differentiation.

Regarding to the mechanisms of PTHrP, some studies demonstrated that PTHrP bound to PTHR1 and then activated the intracellular adenylate cyclase-cyclic adenosine monophosphate-PKA and PLC-cell calcium ion-PKC signaling pathways (Ko et al., 2001). PTHrP also stimulated mesenchymal stem cells differentiating into osteoblasts in the development of jaw, and its regulatory mechanism was mainly mediated by PKA and PKC pathway (Zhao et al., 2002).

In this study, PTHrP-induced stem Leydig cell differentiation was reversed by PKA inhibitor (H-89) and PKC inhibitor (U73122; Figure 1F). These data indicate that PTHrP promotes stem Leydig cell differentiation via both PKA and PKC pathways. In the PKA pathway, the CREB/p-CREB is the eventually transcriptional regulator for gene expressions. Indeed, testicular injection of PTHrP up-regulated CREB/p-CREB levels, suggesting that a PKA-CREB pathway is involved.

In the present study, we demonstrated that *in vitro* treatment of PTHrP up-regulated *Star, Cyp17a1*, and *Hsd17b3* levels. Both rat and mouse *Star* promoter contained CREB regulatory region and cAMP-PKA signaling could up-regulated *Star* expression via CREB/p-CREB (Manna et al., 2002; Clem et al., 2005). Therefore, PTHrP could up-regulate *Star* via PTH1R-cAMP-PKA-CREB/p-CREB signal. Interestingly, at 1,000 pg/ml, PTHrP did not affect *Star* level *in vitro*. The exact mechanism is still unclear. One possible speculation could be the other signaling pathways of PTHrP contributing to this change. CYP17A1 protein was up-regulated after 1,000 pg/ml PTHrP treatment, suggesting that CYP17A1 is the sensitive protein responding to *in vitro* PTHrP treatment. The up-regulation of CYP17A1 will lead to the increased production of testosterone and the loss of CYP17A1 could block the testosterone biosynthesis. How PTHrP selectively up-regulates several gene expression levels is not known.

There is a little discrepancy in the gene expression regulation between *in vitro* and *in vivo* PTHrP treatment. For example, *in vitro* PTHrP mainly up-regulated *Star, Cyp17a1*, and *Hsd17b3* levels, while *in vivo* PTHrP mainly up-regulated *Lhcgr* and *Cyp11a1* levels. The reason is still unclear. Possible explanations are the doses of PTHrP and treatment regimen. Firstly, in the *in vitro* study, the highest concentration of PTHrP was 1 ng/ml, while in the *in vivo* study the highest concentration of PTHrP was 100 ng/testis (about 100 ng/ml). Secondly, PTHrP continuously exposed to cells *in vitro* while it was injected once a day *in vivo*. PTHrP up-regulated CYP11A1 level *in vivo* without affecting CYP11A1 positive cell number. Our previous study showed that when progenitor and immature Leydig cells differentiated into adult Leydig cells, the CYP11A1 protein or enzyme activity level per cell was up-regulated (Guo et al., 2013). This indicates that CYP11A1 protein level per cell is increased and this means that Leydig cells are differentiated into cells at the more mature stage. Although the exact amount of PTHrP in the rat testis is still unknown, PTHrP is mainly expressed in Sertoli cells in rat fetal and neonatal testes, and it is primarily expressed in Leydig cells in adult testes (Campos et al., 1991). In our *in vitro* study, we clearly demonstrated that PTHrP was effective to stimulate the differentiation of Leydig cells at the lower concentrations, suggesting that PTHrP may play a physiological role in Leydig cell development.

There are several factors that play roles in the commitment of stem Leydig cells into the Leydig cell lineage. Apparently, LH is not the factor to regulate the initial commitment, because stem Leydig cells did not contain LHCGR (Ge et al., 2006). Knockout of LHCGR in mice, the progenitor Leydig cells were still formed although the late stage of Leydig cell development was blocked (Lei et al., 2001; Zhang et al., 2001). In this regard, PTHrP could be the factor of the initial commitment of stem Leydig cells. PTHrP is highly secreted by Sertoli cells in the neonatal testis (Campos et al., 1991), where stem Leydig cells were abundantly present (Kilcoyne et al., 2014).

Interestingly, PTHrP did not stimulate the stem Leydig cell proliferation as shown by the facts that no increased EdU incorporation after *in vitro* PTHrP treatment (Supplementary Figure 1) and no increased PCNA-positive Leydig cells after *in vivo* PTHrP treatment (Figure 7) were found. Indeed, in another study using the similar treatment regimen by dbutyryl cyclic AMP, which mimics the endogenous cAMP and stimulates stem Leydig cell differentiation but suppresses EdU incorporation (Li et al., 2016). Since PTHrP is involved in cAMP-PKA-CREB signaling, the present study indicates that the main function of PTHrP in the stem Leydig cell is promoting its differentiation.

## Conclusion

PTHrP stimulated stem Leydig cell differentiation via PKA and PKC signaling pathways without affecting its proliferation. PTHrP regulated stem Leydig cell differentiation by up-regulating several steroidogenic enzyme (*Cyp11a1, Cyp17a1*, and *Hsd17b3*) gene expression. PTHrP could become a drug to stimulate Leydig cell development for therapeutic uses.

## Author contributions

QL and R-SG designed the research project. TS, YW, HL, LC, JL, XC, XiaojL, XiaohL, and LL conducted the research; QL and R-SG had primary responsibility for the final content of the manuscript and wrote the manuscript; TS and R-SG analyzed data or performed statistical analysis and all authors read and approved the final manuscript.

### Conflict of interest statement

The authors declare that the research was conducted in the absence of any commercial or financial relationships that could be construed as a potential conflict of interest.

## Supplementary material

Figure S1 PTHrP does not stimulate EdU incorporation into stem Leydig cells *in vitro*. Seminiferous tubules were incubated with increasing concentration of PTHrP (0, 10, 100, and 1,000 pg/mL) for 5 days. **(A)** the control; **(B)** 1,000 pg/mL PTHrP; EdU labeled cell shows green staining in the nucleus (White arrow). **(C)** Seminiferous tubules were incubated with increasing concentration of PTHrP (0, 10, 100, and 1,000 pg/mL) for 7 days and then the tubules were switched into the medium containing ITS+LH+LI for 14 days being induced into the Leydig cells to produce testosterone. Mean ± SEM, *n* = 6. No significance was found in the testosterone levels between each PTHrP group and control group.
