# Supplementary figures and images for: Parathyroid Hormone-Related Protein Promotes Rat Stem Leydig Cell Differentiation
Source: Front Physiol. 2017 Nov 13;8:911. doi: 10.3389/fphys.2017.00911 (PMC5693895; doi:10.3389/fphys.2017.00911)

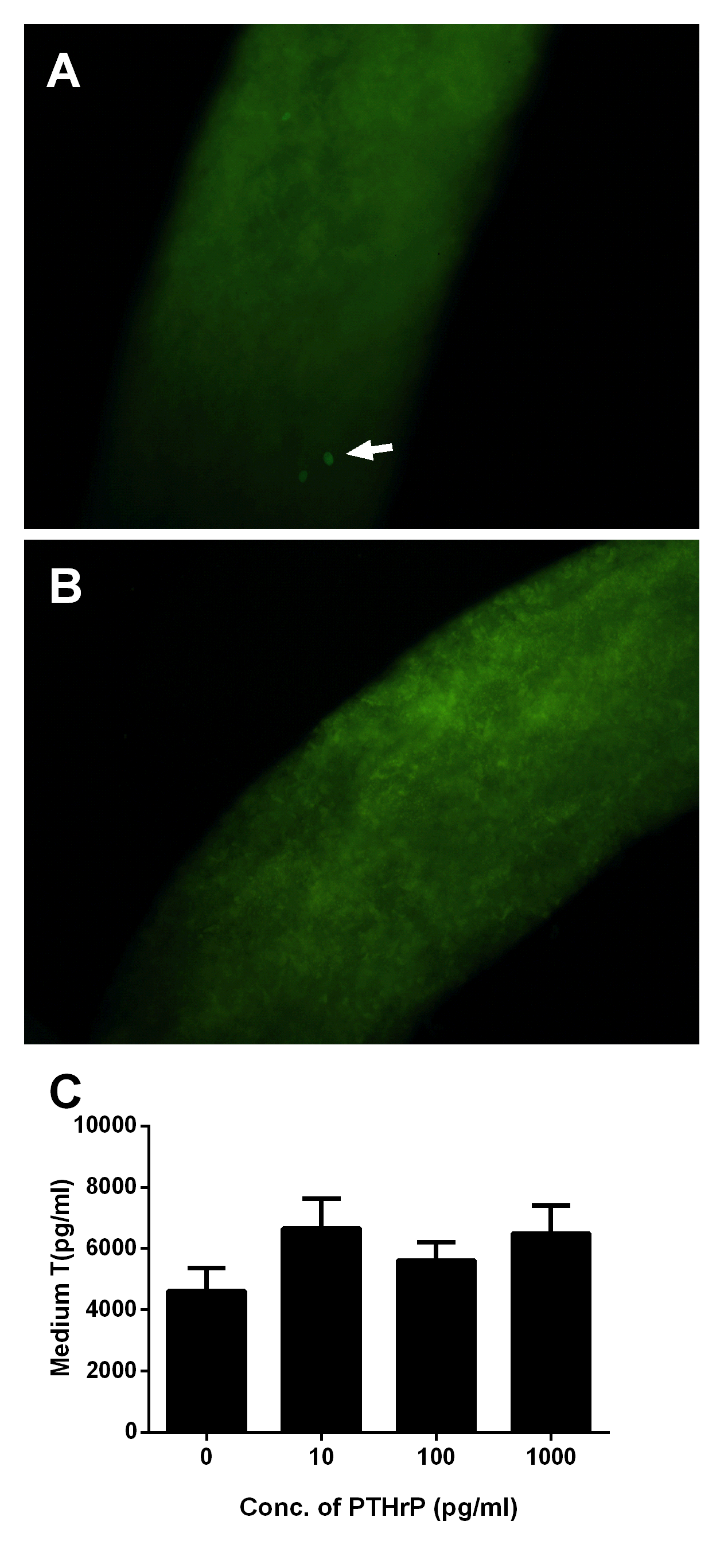

Supplement: Figure S1 — PTHrP does not stimulate EdU incorporation into stem Leydig cells in vitro. Seminiferous tubules were incubated with increasing concentration of PTHrP (0, 10, 100, and 1,000 pg/mL) for 5 days. (A) the control; (B) 1,000 pg/mL PTHrP; EdU labeled cell shows green staining in the nucleus (White arrow). (C) Seminiferous tubules were incubated with increasing concentration of PTHrP (0, 10, 100, and 1,000 pg/mL) for 7 days and then the tubules were switched into the medium containing ITS+LH+LI for 14 days being induced into the Leydig cells to produce testosterone. Mean ± SEM, n = 6. No significance was found in the testosterone levels between each PTHrP group and control group. [file Image1.TIF]
