# Supplementary material for: Parathyroid Hormone-Related Protein Promotes Rat Stem Leydig Cell Differentiation
Source: Front Physiol. 2017 Nov 13;8:911. doi: 10.3389/fphys.2017.00911 (PMC5693895; doi:10.3389/fphys.2017.00911)
Supplement: Supplementary file 3 [file Table2.DOCX]

**Supplementary Table S2. Antibodies**

| **Antibody** | **Species** | **Vendor (City, State, catalogue)** | **Dilution** | |
| --- | --- | --- | --- | --- |
|  |  |  | **WB** | **HS** |
| β-Actin | rabbit | Cell Signaling Technology (Danvers, MA, 12620) | 1:1000 | ND |
| LHCGR | goat | Santa Cruz (Santa Cruz, CA, sc-26343) | 1:1000 | ND |
| CYP11A1 | rabbit | Santa Cruz (Santa Cruz, CA, sc-18043) | 1:1000 | 1:200 |
| CYP17A1 | rabbit | Santa Cruz (Santa Cruz, CA, [sc-66850](https://www.scbt.com/scbt/product/cyp17a1-antibody-m-80?requestFrom=search)) | 1:1000 | ND |
| 17β-HSD3 | rabbit | Abcam (San Francisco, CA, ab126228) | 1:1000 | ND |
| 11β-HSD1 | rabbit | Abcam (San Francisco, CA, ab39364) | 1:2000 | 1:200 |
| Scarb1 | rabbit | Abcam (San Francisco, CA, ab52629) | 1:1000 | ND |
| CREB | rabbit | Abcam (San Francisco, CA, ab32515) | 1:500 | ND |
| p-CREB | rabbit | Abcam (San Francisco, CA, ab32096) | 1:5000 | ND |
| STAR | rabbit | Abcam (San Francisco, CA, ab58013) | 1:1000 | ND |
| PCNA | mouse | Abcam (San Francisco, CA, ab29) | ND | 1:50 |
| SMA | mouse | Sigma Aldrich (Saint Louis, A2547) | ND | 1:200 |

ND = Not detected; WB = Western blot; HS = Histochemical staining.
