# Supplementary material for: Parathyroid Hormone-Related Protein Promotes Rat Stem Leydig Cell Differentiation
Source: Front Physiol. 2017 Nov 13;8:911. doi: 10.3389/fphys.2017.00911 (PMC5693895; doi:10.3389/fphys.2017.00911)
Supplement: Supplementary file 4 [file Table3.DOCX]

Supplementary Table S3. The body weight and testis weight of rats after PTHrP treatment.

| Parameters | PTHrP doses (ng/testis) | | |
| --- | --- | --- | --- |
|  | 0 | 10 | 100 |
| Body weight (g) | 432.6±14.37 | 383.3±18.56 | 405.0±13.99 |
| Testis weight (g) | 1.15±0.06 | 1.19±0.09 | 1.17±0.05 |

Mean ± SEM, n = 6.
